# Supplementary material for: Low circulating adropin concentrations predict increased risk of cognitive decline in community-dwelling older adults
Source: GeroScience. 2023 May 26;46(1):897–911. doi: 10.1007/s11357-023-00824-3 (PMC10828274; doi:10.1007/s11357-023-00824-3)
Supplement: Supplementary file 1 — Supplementary file1 (DOCX 20 KB) [file 11357_2023_824_MOESM1_ESM.docx]

**Supplemental Table 1.** Plasma concentrations of inflammatory markers (soluble tumor necrosis factor receptor, sTNFR-1; monocyte chemoattractant protein 1, MCP-1; interleukin 6, IL-6, C-reactive protein, CRP) and cellular stress (plasma growth differentiation factor 15, GDF-15). The data shown were measured at V3 and V5 and are presented as estimated marginal means±SE adjusted for age. P values for covariates less than 0.05 are shown in *italics*.

*(table S1)*

| **Metric** | **Sex** | **Adropin tertile** | | | | **P value** |
| --- | --- | --- | --- | --- | --- | --- |
|  |  | ***(ALL)*** | **1st** | **2nd** | **3rd** |  |
| sTNFR-1  (pg/mL) | F  M  ALL | *1334±31*  *^(240)^*  *1447±36*  *^(167)^*  *1390±24*  *^(407)^* | 1283±57  ^(68)^  1493±65  ^(52)^  1388±43  ^(120)^ | 1374±52  ^(84)^  1428±62  ^(57)^  1401±40  ^(141)^ | 1345±50  ^(88)^  1418±62  ^(58)^  1382±40  ^(146)^ | *Age, P<0.001*  Sex, P<0.05 |
| MCP-1  (pg/mL) | F  M  ALL | *248±6*  *^(240)^*  *242±7*  *^(167)^*  *245±5*  *^(407)^* | 244±11  ^(64)^  235±13  ^(52)^  239±9  ^(120)^ | 236±10  ^(84)^  229±12  ^(57)^  233±8  ^(141)^ | 265±10  ^(88)^  262±12  ^(58)^  263±8  ^(146)^ |  |
| IL-6  (pg/mL) | F  M  ALL | *5.65±1.19*  *^(240)^*  *3.97±1.42*  *^(167)^*  *4.81±0.93*  *^(407)^* | 9.36±2.23  ^(68)^  3.90±2.55  ^(52)^  6.63±1.69  ^(120)^ | 4.02±2.01  ^(84)^  3.69±2.43  ^(57)^  3.86±1.58  ^(141)^ | 3.57±1.96  ^(88)^  4.31±2.42  ^(58)^  3.94±1.56  ^(146)^ |  |
| CRP  (mg/L) | F  M  ALL | *3.07±0.32*  *(^245)^*  *2.93±0.39*  *^(163)^*  *3.00±0.25*  *^(408)^* | 2.75±0.55  ^(80)^  2.30±0.66  ^(56)^  2.52±0.43  ^(136)^ | 3.50±0.53  ^(87)^  2.87±0.67  ^(55)^  3.18±0.43  ^(142)^ | 2.96±0.56  ^(78)^  3.62±0.69  ^(52)^  3.29±0.44  ^(130)^ |  |
| GDF-15  (pg/mL) | F  M  ALL | *1198±32*  *^(239)^*  *1438±38*  *^(167)^*  *1318±25*  *^(406)^* | 1225±59  ^(68)^  1571±68  ^(52)^  1398±45  ^(120)^ | 1183±54  ^(83)^  1382±64  ^(57)^  1283±42  ^(140)^ | 1184±52  ^(88)^  1361±64  ^(58)^  1273±41  ^(146)^ | *Age, P<0.001*  Sex,  P<0.001 |
